# Supplementary material for: Peptide-Mediated Targeted Delivery of Aloe-Emodin as Anticancer Drug
Source: Molecules. 2022 Jul 19;27(14):4615. doi: 10.3390/molecules27144615 (PMC9320513; doi:10.3390/molecules27144615)
Supplement: Supplementary file 1 [file molecules-27-04615-s001.zip › molecules-1776133-supplementary.pdf]

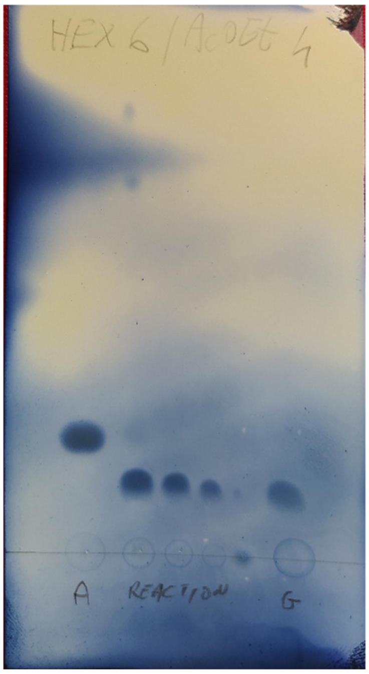

Figure S1. TLC of synthesis steps of (3) A: Aloe-emodin (2) (Rf=0.21), Reaction: reaction mixture after 8 hours, G: monoglutaril derivative (**3**) (Rf=0.12).

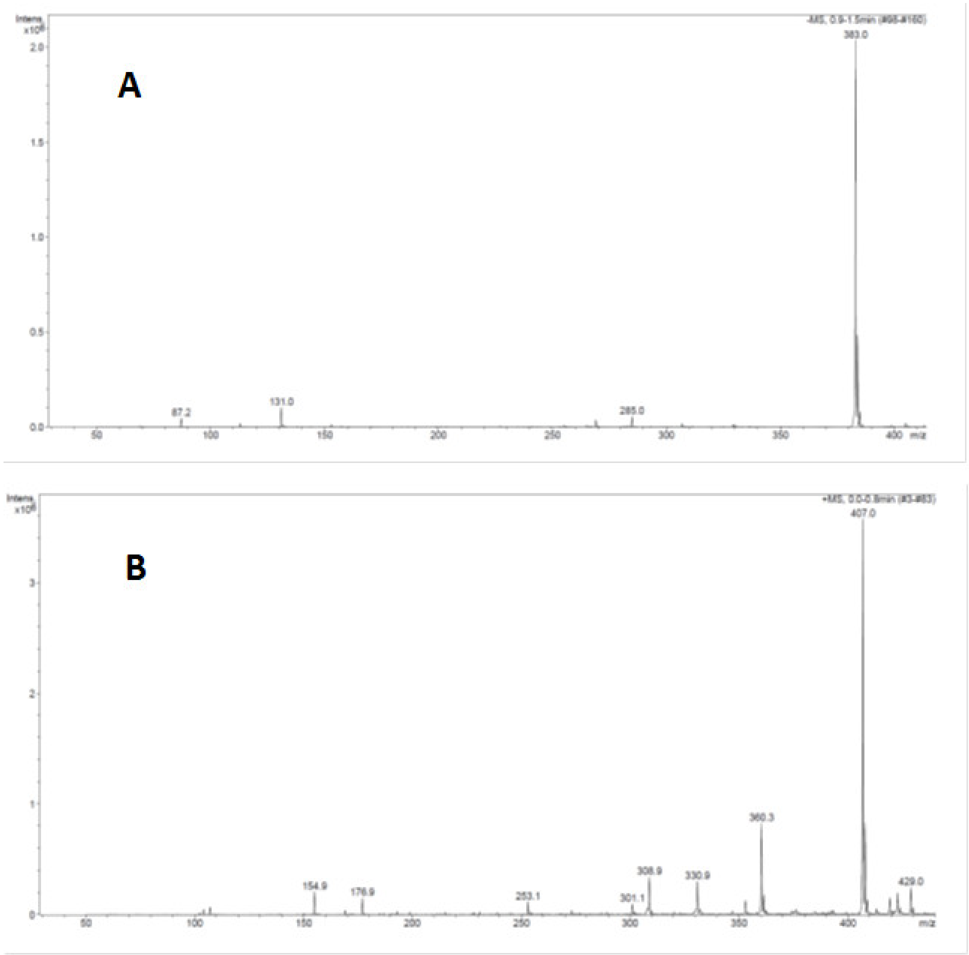

Figure S2. Mass spectra (ESI detector) of compound **3**: negative ions (**A**); positive ions (**B**).
